# Application of Hydroxytyrosol in the Functional Foods Field: From Ingredient to Dietary Supplements

**DOI:** 10.3390/antiox9121246

**Published:** 2020-12-08

**Authors:** Andreia F. R. Silva, Daniela Resende, Mariana Monteiro, Manuel A. Coimbra, Artur M. S. Silva, Susana M. Cardoso

**Affiliations:** LAQV-REQUIMTE, Department of Chemistry, University of Aveiro, 3810-193 Aveiro, Portugal; afrs@ua.pt (A.F.R.S.); danielaresende@outlook.com (D.R.); marianaicnamonteiro@gmail.com (M.M.); mac@ua.pt (M.A.C.); artur.silva@ua.pt (A.M.S.S.)

**Keywords:** phenol, olive oil, by-products, table olives, antioxidant, functional ingredient, novel foods

## Abstract

Hydroxytyrosol (HT) is an amphipathic functional phenol found in the olive tree, both in its leaves and fruits, in free or bound forms, as well as in olive oil and by-products of olive oil manufacture. The European Food Safety Authority recommends regular consumption of HT due to its several beneficial effects on human health, which are closely associated to its antioxidant activity. These reasons make HT an excellent candidate for application as a functional ingredient in the design of novel food products. Patents already exist for methodologies of extraction, purification, and application of HT in supplements and food products. The present review discusses the impact of HT incorporation on food properties and its effects on consumers, based on relevant data related to the use of HT as a functional ingredient, both as a pure compound or in the form of HT-rich extracts, in various food products, namely in edible oils, beverages, bakery products, as well animal-based foods such as meat, fishery and dairy products.

## 1. Introduction

The assumption that “diet is the best medicine” embraces the valorization of food ingredients and, in particular, of health-promoting bioactive compounds. Hydroxytyrosol (HT) is a dietary constituent that has received much attention in the last decade due to the many benefits of the ingestion of virgin olive oil, relative to antioxidant capacity and properties against cardiovascular diseases. This phenolic is recognized as safe by the European Food Safety Authority (EFSA) and Food and Drug Administration (FDA). Based on the opinion of the EFSA, in 2017, the European Commission (EC) authorized the placing on the market of HT as a novel food ingredient under Regulation (EC) No 258/97 [1,2], which assumes its use for the general population, excluding children under the age of three years, pregnant women, and lactating women [3]. To promote its widespread consumption, HT has been integrated into the concept of functional foods, i.e., a natural-based food product provider of health benefits beyond the primary nutrition value, which has exponentially increased over the last few years.

HT, i.e., 3,4-dihydroxyphenylethanol, is an amphipathic phenol with the potential to be used as an ingredient in functional foods. In fact, it is stated by EFSA opinion of 2011 and EC regulation 432/2012 that HT is the only “food” officially accepted to have an obvious health effect when consumed regularly with the prerequisite to contain at least 5 mg/20 g oil of HT and related compounds [4]. The main dietary sources of HT include olive oil and table olives, in free and/or bound forms, although this is also present in olive leaves [5] and in lower amounts in grapes [6] and wine [7]. Its formation occurs during the ripening process of olives, as well as during the preparation of table oils and oil storage, by hydrolysis of oleuropein [8]. In olives, this may be a consequence of the acidic conditions (resulting from the brining of fruits), the activity of endogenous olive enzymes [9] or from the fermentation of lactic acid bacteria [10], whereas in wine, HT results from the conversion of tyrosol through alcoholic fermentation, or enzymatic oxidation by polyphenol oxidase present in the fruits [6,11]. Note that oleuropein is composed of a glycosylated ester of elenolic acid with HT, which makes it suitable to be hydrolyzed by β-glucosidase enzymes. However, oleuropein and HT share catecholic structures, conferring them similar antioxidant activity [9]. 

According to the EFSA, the average HT content, in mg/kg of product, ranges from 3.5–7.7 in olive oils and it is approximately 556 and 660 in green and black olives, respectively [1]. Thus, considering the dietary data from the European Union, it is estimated that the mean values of HT consumption in adults vary between 0.15–4.0 and 19–185 µg of HT/kg body weight (bw) per day, as a result of olive oil and table olives intake, respectively [1], far from the daily recommended intake. In addition, table olives and olive oil are not consumed by everyone, which relays the importance of the incorporation of HT in other types of products. 

The health-promoting abilities of dietary HT are dependent on metabolism, bioaccessibility and bioavailability issues. Similar to most phenolic compounds, no salivary digestion was reported for HT, possibly due to its inhibitory effect on α-amylase [12]. In the gastric phase, most of HT is released from the food matrix and remains stable [13] while metabolization is triggered in the intestinal phase, due to the basic pH conditions and the presence of enzymes in enterocytes. The compound is absorbed by the enterocytes of the epithelium and reaches the liver through the hepatic portal vein, where metabolism continues. Afterward, HT and its metabolites are further excreted into systemic circulation or can be reabsorbed by the intestine through bile [14]. During this process, it is gradually converted into other metabolites such as 3,4-dihydroxyphenylacetaldehyde, 3,4-dihydroxyphenylacetic acid (DOPAC) and 4-hydroxy-3-methoxyphenylacetic acid (or homovanillic acid), which can be detected in human plasma [15]. Hydroxytyrosol acetate is another metabolite often detected in human plasma, resulting from the alkaline hydrolysis and acetylation of HT in the lumen [16,17]. For the study of the health benefits of HT consumption, it is of utmost relevance to know which metabolites are generated once, similar to HT, some of them exert antioxidant activity, due to the hydroxy group in the 3,4-ortho position [18], such as DOPAC [19] and homovanillic acid [20]. Hydroxytyrosol acetate exhibits less antioxidant activity than HT due to the acetate group of the ester that may hide the scavenging effect of the hydroxyl groups by intra- or intermolecular hydrogen bonding [21]. Still, it inhibits platelet aggregation in rats [22], an important cardiovascular comorbidity. Otherwise, 3,4-dihydroxyphenylacetaldehyde is a reactive and toxic metabolite [23], which may interfere in the bioactivity of the remaining compounds. 

As HT is well absorbed and rapidly metabolized [24], its concentrations in plasma can be very low, although other factors such as the vehicle of ingestion may also interfere. For example, after ingesting 25 mL of extra olive oil with 61.4 µg HT/g olive oil [25] or 50 mL of olive oil extract that contains 6.0 µg HT/mL extract, along with bread [14], the maximum HT concentration detected in human plasma was 0.004 µg/L after 15 min of intake and 25.8 µg/L after 32 min, respectively. Additionally, the input of a single dose of 2.5 mg of HT/kg bw in aqueous solution reached 169 µg HT/L 13 min upon ingestion but quickly decreased to undetectable levels after 1 h [26]. Differences between the amount ingested and the levels detected in plasma support the hypothesis that the main part of HT has undergone the first-pass metabolism in great extension and was converted into other metabolites [14]. Moreover, in the study of Tuck and coworkers [27], the authors demonstrated that the estimated HT plasma concentration was significantly reduced if HT was administered to rats as an aqueous solution (solution of radiolabelled HT, 12 mCi/mmol H^3^-HT) when compared to a close amount administered as an olive oil solution (17 mCi/mmol). In addition, López de las Hazas and collaborators [24] noticed that the absorption of HT could be improved after dietary supplementation with oleuropein. Overall, it is crucial to further understand the extension of HT metabolization and mechanisms of action in order to establish HT dose effects. In addition, the possible contribution of other phenols such as tyrosol (which may be converted in the human body into HT and vice-versa) to the levels of bioavailable HT and its claimed bioactivities should be clarified [28].

## 2. Antioxidant Properties of HT

HT is claimed to exert many bioactive properties, including antioxidant, anti-inflammatory, anticancerogenic, neuroprotector, immunomodulatory, cardioprotective, antidiabetic, cytoprotective, antimicrobial, antiviral, endothelial and vascular regulatory, and skin protective properties [15,29,30,31]. Among these, antioxidant abilities are undoubtedly one of the most relevant properties for application of HT in the food industry, not only because they can impact on the quality of the fortified food products, but also in parameters related to consumers’ health. Regarding prevention of cardiovascular diseases, in addition to its antioxidant capacity, HT was also claimed to inhibit platelet aggregation, chronic cardiac toxicity, the expression of proteins related to ageing, as well as attenuation of the metabolic alterations of glucose, triglyceride and total cholesterol [29].

The mechanisms of action of the antioxidant activity of HT include the breakdown of peroxidation chain reactions, such as lipid peroxidation, the scavenging of free radicals, e.g., radical superoxide (O_2_^•−^), hydrogen peroxide (H_2_O_2_) and hypochlorous acid (HOCl), and the prevention of metal ions catalyzing the production of reactive oxygen species (ROS) [15,29]. These properties are due to its catechol group [18] and its ability to act as a chelate of the intracellular iron [32]. 

The antiradical potential of HT or of HT-rich extracts was confirmed by distinct authors through in vitro assays [33,34,35], albeit contradictory observations were reported in some cases. Visioli et al. [33] reported the intense radical-scavenging activity of HT and oleuropein towards 2,2-diphenyl-1-picrylhydrazyl radical (DPPH^•^), which was superior to those of 2,6-di-*t*-butyl-4-methylphenol (BHT) and vitamin E, and the ability to eliminate O_2_^•−^ and HOCl, in equivalent or higher extension as the reference vitamins E and C. However, in another study, despite both oleuropein and HT being potent hydroxy radical (OH^•^), O_2_^•−^, and peroxynitrous acid (ONOOH) scavengers, none of these compounds showed potential to sequester HOCl or H_2_O_2_ [35]. Moreover, O′Dowd et al. [34] reported that HT had no potential to scavenge O_2_^•−^ whereas it was effective towards H_2_O_2_. 

Despite the inconsistency described for the HT scavenging ability in chemical models, cellular studies support its high antioxidant capacity. As shown by Peng and coworkers [36], in rat adrenal pheochromocytoma cells, the pre-treatment of the cells with this phenolic compound at non-toxic concentrations (1–50 μM) remarkably reduced toxic effects induced by H_2_O_2_ and 6-hydroxydopamine, including amelioration of cellular viability to values above 80%, decreasing of ROS production and prevention of the loss of cellular thiols, which are essential in the intracellular redox balance. Another study performed with kidney cell lines of distinct animals (Madin–Darby canine MDCK, a pig kidney LLC-PK1, and a rabbit kidney RK 13) revealed that 10 µM HT effectively prevented cytotoxicity and the increment of ROS levels induced by ochratoxin-A (a toxic able to cause kidney disorders) [37]. Similarly, in human hepatocarcinoma HepG2 cells oxidized by *t*-butyl hydroperoxide, the pre-treatment with 10–40 μM of HT completely prevented the cell damage and maintained glutathione (GSH; a thiol antioxidant) levels [38]. In addition, cell damage prevention in apoptosis-responsive-cellular models (human monocytoid U937 and murine proliferating muscle C2C12 myoblasts) exposed to H_2_O_2_ was also noticed with pretreatments of 20 μM of HT or its ester HT laurate [39], and with 0.05 mM and 0.1 mM of HT in Jurkat cells (clone E6-1, peripheral human blood), 30 min before H_2_O_2_ exposure [32]. Notably, in the work of Rietjens et al. [35], the authors demonstrated that the exposure of low-density lipoproteins (LDL) of healthy human volunteers’ blood to 5 µM HT prevented copper (II) sulphate-induced oxidation increased the lag time of conjugated dienes formation (products of oxidation) from 30 to 240 min. The ability to hamper lipid oxidation is extended to HT-rich extracts, as previously demonstrated, e.g., for a purified extract from the solid olive residue (the base of the supplement Oleaselect^TM^ (Milan, Italy)). This extract, containing HT and other phenols, effectively restored the cells and reduced the lipid oxidation in haemolysis-induced cellular rat erythrocytes and endothelial cells [40].

The antioxidant effect of HT also occurs through its ability to stimulate the expression and the activity of antioxidant enzymes. In fact, HT has been claimed to modulate the gene expression of antioxidant enzymes such as catalase (CAT), superoxide dismutase (SOD) and glutathione peroxidase (GPx), which are key regulators of oxidative stress, and the expression of inflammation transcription factors [30,41,42]. According to Zhu et al. [42], at a concentration of 100 μmol/L, HT effectively regulated the transcription of the nuclear factor erythroid 2 (Nfr2) in acrolein-stimulated human retinal pigment epithelial cells (ARPE-19 cells), activating phase II detoxifying enzymes, such as γ-glutamyl cysteine ligase (produces reduced GSH), GPx, nicotinamide adenine dinucleotide phosphate (NADPH)-quinone-oxidoreductase, heme-oxygenase, SOD, peroxiredoxin and thioredoxin. HT also activated peroxisome proliferator-activated receptor coactivator 1α (PPARGC1α), which enhanced mitochondria biogenesis. As a result, it blocked the oxidative damage induced by acrolein and, therefore, the degeneration of these cells. Additionally, Giordano and Visioli [43] reported that HT modulated GSH-driven antioxidant enzymatic machinery of mice pre-adipocyte cells (murine 3T3-L1), thus mitigating the H_2_O_2_-induced alterations regarding the reduced/oxidized GSH ratio, an oxidative stress indicator.

Antioxidant-related health effects of HT are supported by animal studies. Granados-Principal et al. [44] demonstrated that the administration of 0.5 mg HT/kg, five days/week for six weeks in Sprague-Dawley rats with breast cancer, mitigated the cardiotoxicity caused by doxorubicin (a chemotherapy drug), reduced ROS production and restored the redox chain in mitochondria. In another work, González-Santiago and coworkers [45] reported that the supply of 4 mg of HT/kg bw to hyperlipidemic male New Zealand rabbits for one month led to an improvement in the antioxidant status and the blood lipid profile of the animals, as well as to a decrease in the levels of total cholesterol and total triglycerides. Additionally, the same study noticed a reduction in the size of arteriosclerosis lesions in a histological cross-section of the aortic arch, a consequence of the hyperlipidemic rabbits. Furthermore, the supply of 40–80 mg HT/kg bw to lipopolysaccharide (LPS)-induced inflammation Balb/c mice subjected to different time points of administration caused an overall increment of the plasma antioxidant power, prevented the LPS-induced DNA damage and decreased the levels of several pro-inflammatory markers such as cyclooxygenase-2 and tumor necrosis factor-α (TNFα) [46]. Pan and coworkers [47] also highlighted a decrease in the levels of TNFα and other inflammatory proteins (interleukin-6 and macrophage inflammatory protein 2) in the liver tissues of male mice Balb/c and an increase in CAT and SOD activity after HT treatment (1 or 10 mg HT/kg), overall preventing ischemia/reperfusion injury of the liver. In addition, Feng et al. [48] reported that HT was able to regulate dynamic mitochondrial remodeling and enhance antioxidant enzyme activity of the respiratory chain in the muscle of rats subjected to strenuous physical exercise. 

In vivo studies performed with HT-rich extracts also support HT antioxidant-related capacities. In a study conducted by Jemai et al. [49], the authors used olive leaf extract (OLE) and its corresponding acid and enzymatic hydrolysate extracts (1.4 g HT/100 g and 3.82 g HT/100 g dry weight, respectively) to supplement the cholesterol-rich diet of rats over sixteen weeks, having shown that these promote the activity of hepatic antioxidant enzymes and a simultaneous decrease in the levels of total cholesterol, triglycerides and LDL, compared to rats fed with a non-supplemented cholesterol diets. Additionally, the feeding of diabetic rats with a pellet diet fortified with a purified-HT extract from olive milled waste origin (20 mg HT/kg bw) was shown to increase the activity of SOD, CAT and GPx in the liver and kidney, as well as other enzymes that catalyze the phosphorylation of glucose. As a result, the glucose level in plasma decreased by 55% [50]. Moreover, in an European clinical trial (EUROLIVE study, Effects of Olive Oil Consumption on Oxidative Damage in European Populations), the daily intake of virgin olive oil (VOO), with 366 mg of phenolic content/kg, over three weeks was demonstrated to increase high-density lipoprotein (HDL) content while also reducing lipid oxidation in human volunteers [51]. This is consistent with other pre-clinical studies that remarked the ability of HT in reducing thrombogenic, platelet aggregation factors and markers of inflammation at a cellular level [15,29], thus inhibiting arteriosclerosis, among other comorbidities [29,31].

## 3. Applicability of Hydroxytyrosol in Food Products

### 3.1. Consumption Recommendations 

The minimum recommended HT amount (including its derivatives) for manutention of cardiovascular health by the EFSA and FDA panel [1,2] reinforces the necessity to incorporate HT in other food products. As an integrated part of a food product, the EFSA has established HT concentrations for fishery products and vegetable oils, as well as for margarine, of 215 mg/kg and 175 mg/kg, respectively. For other types of food, the amount of HT is suggested to be adjusted, taking into account the HT content in the background diet [1]. Moreover, according to the FDA, final concentrations should comprise between 8 and 21 mg/kg for energy drinks and vegetables/fruits beverages, respectively; 167 to 250 mg/kg for snacks and baked goods; 44 to 333 mg/kg for sauces, condiments, fat and oil products and, finally, about 1.1 g/kg for meat, poultry and fish coating mixes [2]. 

So far, the HT toxicological data are mainly based on cell and animal studies. Among them, the study of D’Angelo and coworkers [52] represented a hallmark, with no adverse effects (NOAEL) being registered upon the induction of acute toxicity in rats by injection of a single dose of 2 g of HT/kg bw. Afterward, subchronic toxicity studies with oral gavage administration through a daily dose of aqueous olive pulp extract at levels of 500, 1000 or 2000 mg/kg [53], as well as 5, 50 or 500 mg HT/kg bw per day [54], did not cause any adverse effects. Auñon-Calles and his collaborator [54] assumed the lowest observed adverse effect level at 500 mg of HT/kg bw per day and a NOAEL of 250 mg/kg bw per day. The only difference noted was salivation before and after administration in all animals, which the authors attributed to the bitter taste of HT and the oily and dense formulation. Considering these studies, the EFSA panel recommended a maximum daily HT intake of 100 mg/kg body weight per day for children and 200 mg/kg body weight per day for adolescents, adults and elderly [1].

### 3.2. Application of HT in the Functional Food Market

As for other natural bioactive compounds, obtaining pure HT and its use in food products is hardly considered economically viable. Therefore, a variety of approaches to meet market requirements for a lower cost HT, including its exploitation from natural sources or the use of biocatalysis, have been proposed [55]. Part of this solution involves the use of by-products. In fact, the cheapest sources of HT comprise olive pomace, olive mill wastewaters (OMWW), and olive leaves, from which the obtention of HT-rich extracts or pure HT requires the combination of solvent extraction, membrane filtration technology and stabilization by lyophilization or spray-drying [56]. Thus, it is possible to find many patented adaptations of the olive pressing systems, extraction procedures [57,58,59] and purification methods [60], aiming at the full use of these resources [61], and at the promotion of circular economy processes, through the usage of olive oil by-products for the development of the extracts. 

#### Supplements and Patented HT-Rich Extracts

HT-rich extracts are widely marketed as food supplements or healthy ingredients. Among them, Hidrox (CreAgri, Hayward, CA, USA) is a formulation of olive polyphenols rich in HT obtained from vegetative waters, certified as Generally Recognized as Safe (GRAS) and patented (patent EP06025262A). It is used to formulate other supplements such as the OLIVACTIV™ (20–35 weight % of HT), the OLEASELECT™ (total content of HT of ≥1.5 weight %), the OLIVE(OLEA)DRY, i.e., a powder containing from 22 to 24 g of HT per kg, and Prolivols (20 mg of HT per g) [62]. Hytolive (Genosa, Spain) is a patented olive extract [63], which was previously shown to be effective in reducing inflammation among early-stage breast cancer patients [64]. Phenolea^®^ (Phenofarm, Scandriglia, Italy) is an active complex also derived from a natural standardized olive pulp extract through a patented mechanical process, in which HT accounts for 30% of all phenols. Evidence has shown that its administration to mice modulates the level of cytokines, having a role in the process of inflammation [65]. Moreover, Aponte et al. [66] used Phenolea^®^ as a source of HT to produce oral granules for co-delivery of *L. plantarum* and a standardized OLE. Through in vivo data, they noticed that co-administration of live *L. plantarum* bacteria with the olive phenol-containing extract provided higher amounts of bioavailable HT compared to the extract alone. Another example is the Oleaselect^®^ (Indena, Milan, Italy), obtained by a patented process (patent US 6358542B2) from olive-based starting materials, including olives, olive pulps, olive oil, and wastewater from olive oil manufacturing, and claimed to achieve an extract with antioxidant activity four to five times higher than the hydroalcoholic extract of solid olive residue [40]. Mediteanox^®^ (Euromed S.A., Barcelona, Spain) is another registered supplement. Notably, oral administration of three capsules of supplement that contains a combined 3.3 mg of HT from Mediteanox^®^ and 65 mg of punicalagin from Pomanox^®^ (a pomegranate natural extract, Euromed S.A., Barcelona, Spain), per day for eight weeks, significantly improved the endothelial function and blood pressure, and reduced LDL oxidation in middle-aged subjects [67]. In the case of Mediteanox^®^, the studied dosage gets closer to the minimum daily recommendation (5 mg HT per day).

### 3.3. HT as a Food Ingredient

#### 3.3.1. Plant-Based Products

HT has also been proposed as a functional ingredient in various food matrices, including edible oils [68], beverages, bakery products, as reported in Table 1 [69,70,71,72,73,74,75,76,77,78,79,80,81,82,83,84,85,86,87,88,89,90,91,92,93,94,95].

##### Edible Oils

Edible oils, particularly refined ones, are prone to oxidation [68], but fortification with HT can delay this phenomenon [69,70,71,72,73,74]. The fortification of edible oils has been obtained by the addition of the pure compound or through extracts. OMWW extract (containing 1225.6 mg/L of HT, among other phenols), added to refined olive oils at a concentration of 200 mg/kg, reduced the peroxide values and delayed the oxidation rate, even better than BHT [71]. In addition to HT, this OMWW extract also contained DOPAC, an HT derivative, which could contribute to these effects [71]. Additionally, with OMWW extract, the supplementation of oils for French fries significantly hampered the oxidation of α-tocopherol and the formation of unwanted compounds (e.g. acrolein and hexanal) during the frying process. Curiously, this supplementation increased the number of available sugars and, consequently, the extension of Maillard reactions, resulting in French fries with a more positive taste and better color [73]. In echium oil, which is more susceptible to oxidation than other vegetable oils, 200 mg of HT/kg substantially protected the oil from the oxidation process [69]; however, this was only verified under non-thermal conditions [75]. 

In addition to HT, other compounds that exist in HT-rich extracts may exhibit a synergic effect. This observation was made by Lama-Muñoz and coworkers [72], who reported that a mixture of HT with 3,4-dihydroxyphenylglycol (DHPG) (proportion 2:1) was more efficient in delaying the oxidation of the vegetable oil than the commercial Olivefen^®^. This reinforces the idea that natural antioxidants’ activity is affected by multicomponent foods and varies according to the extract composition.

Nevertheless, regarding the health effects of HT in oils, the main studies focus on olive oil, which is the best natural source of HT through diet. For example, Valls et al. [74] tested a single dose of 30 mL of olive oil supplemented with a phenolic-rich extract from olive cake (with 6.64 mg HT/kg olive oil cake) in hypertensive patients (in pre- and stage 1). They found that the olive oil supplement caused more positive effects on the patient’s endothelial function than the ingestion of a standard virgin olive oil, especially in terms of the decrease in oxidized LDL and blood stimulation flow (reactive hyperemia). Similarly, Farràs et al. [70] evaluated the regular intake of 25 mL per day of distinct olive oil that provided 0.01, 0.12 and 0.21 mg of HT, for three weeks in hypercholesterolemic patients, and they noticed benefits, namely in the improvement of HDL levels and blood plasma antioxidant activity, without increasing the individual’s fat intake. Thus, in addition to the ability of HT to stabilize oily matrices, edible oils fortified with HT may also be suitable to act as a functional food to prevent cardiovascular comorbidities.

At present, there are also several patented applications of HT in edible oils, Más et al. [76] patented fortified edible oils, fortified edible oil-containing products and dietary supplements in the form of soft gel capsules containing fortified edible oils, with increased antioxidant capacity to be used as a source of HT for prevention or treatment of cardiovascular diseases, plaque build-up in the arteries, arterial hypertension and metabolic syndrome [76]. In the same line, Bulbarello and collaborators [77] patented capsules for oral consumption of an edible oil fortified with HT (more than 250 mg HT/kg fortified oil). Moreover, the addition of an extract of bisphenol with more than 60% of HT and a terpene extract with more than 80% of maslinic acid to an extra VOO or olive oil was also patented [78].

##### Beverages

In beverages, wine is valorized as a functional food towards cardiovascular diseases due to its polyphenol content, especially resveratrol and HT, the latter resulting from tyrosol through alcoholic fermentation [7]. However, the use of sulfur additives, such as a sulfur oxide (SO_2_), to prevent the degradation of wine, including oxidation, may induce a range of adverse health effects in sensitive individuals [79]. Thus, efforts have been made to reduce or replace its usage, betting on the use of HT as a promising natural antioxidant alternative [80,81], although the characteristic aroma of HT-rich extracts may hinder its application [82]. In fact, Raposo et al. [80] demonstrated that the use of 80 mg of HT/L in white wine (Sauvignon Blanc) intensified its color when compared to control samples, even after six months of storage in a bottle, but its score was injured with regard to the olfactometric profile. In red wines (Syrah), Raposo and coworkers [82] reported that its fortification with 50 mg of HT/L improved color parameters (namely the red chromatid) and the scent and tasting at bottling. However, after six months of storage, the bottle HT wines were more oxidized than SO_2_ wines, showing new odorant zones from the added product and oxidation. Hence, the authors recommended the combined use of HT with sulfur dioxide, reducing the sulfur content without compromising the wine oxidation.

The application of HT was also demonstrated in fruit juices [83,84] to improve their nutritional properties. In fruit smoothies, the addition of OLE (HT-rich extract) increased their bitterness, but this could be masked if lower concentrations were used (below 20 mg/100 g), or by adding ingredients with known bitterness-masking properties [83]. However, the lower concentrations proposed had lower phenolic content compared to coffee or tea [83]. In an attempt to formulate a functional juice, Larrosa et al. [84] added enzymatically-synthesized HT at a concentration of 1 mg/mL to tomato juice and claimed that 200 mL of this juice would provide 4 mg of HT, which is close to the minimum recommended by the ESFA. In this matrix, the HT was more efficient than a commercial antioxidant (3-t-butyl-4-hydroxyanisole, BHA) in improving antioxidant activity and deaccelerating lipid oxidation (over eight- and three-fold, respectively). Additionally, HT remained stable in the tomato juice matrix at room temperature and light exposure storage conditions for 48 days without affecting the sensory properties (flavor and color) in great extension [84]. 

More recently, Guglielmotti and coworkers [85] incorporated HT as part of brewing to fortify a beer. They used olive leaves as an ingredient of beer, in the form of dry crumbled leaves (1.27 mg of HT/g), infusion (3.43 mg of HT/g), and an atomized extract (5.58 mg of HT/g), that were added near to the boiling phase of brewing, thus promoting the hydrolysis of oleuropein of the leaves to HT. The addition of 10 g/L of olive leaves conferred to the beers a sour/astringent taste and herbal aroma, whereas 5 g/L olive left a more soft and pleasant sensory profile. Both results could be interesting to formulate distinct beers, especially with increased antioxidant capacity and polyphenol content.

Regarding HT’s sensorial impact, efforts have been made to overcome HT’s bitter taste in the products. An example of this is a patented beverage with HT (in the amount of 0.5–50 mg/100 mL) that claims to be easily drinkable due to the presence of ethanol, propylene glycol, and caffeine at a specific rate that disguises the characteristics of the flavor of HT [86].

##### Vegetable-Based Products

Although table olives are a source of HT, their fermentation process often changes and/or reduces their phenolic fraction. Some authors proposed the supplementation with HT or HT-rich extracts to balance this loss (Table 1). In fact, even supplemented with OLE, there was a decrease in the concentration of total phenolics during the fermentation of table olives, but, in the case of HT, it increased upon 120 days in brine and olive flesh samples, achieving in the final of fermentation the levels of 2489 mg/L of HT and 187 mg HT/kg, respectively, with no or only slight changes in their sensory acceptability [88]. These observations were consistent with the work of Schaide et al. [89], who reported that the addition of OLE to table olives increased the levels of HT (1700 mg/kg in olive flesh and 3500 mg/L in brined olive flesh) as compared to control conditions (900 and 2500 mg/L respectively), and provided olives without bitterness after 121 days of fermentation. In line with the previous studies, the sensory acceptability was unchanged in Lalas et al.’s study [87], after the addition of OLE (200 mg HT/kg extract) to table olives fermented for a week, while the HT content increased from 408 to 855 mg/kg in flesh table olives. 

In addition to table olives, the fortification of bean purée, potato purée, and tomato juice with an OMWW extract (0.44, 1.00, 2.25, or 5.06 g extract/kg food product) increased bitterness and intensified the sourness and astringency. Pungency was suppressed in bean purée and perceived at a weak-moderate intensity in potato purée and tomato juice samples at the highest phenol concentration [90]. As table olives are already a natural source of HT, their application should be more explored in other vegetable-based products.

##### Bakery Products

Bakery products are attractive food products for HT supplementation since they are largely consumed by different populational groups of all ages. Among bakery products, a major part of the studies investigated biscuits. For instance, Mateos et al. [91] reported that the human intake of HT-fortified biscuits (30 g biscuits that provide 5.25 mg of HT, after an overnight fast) was able to reduce the levels of oxidized LDL in blood, even though there was no increase in the antioxidant activity in blood serum. In the same line but with a different source of HT, Conterno et al. [92] found that the 90 g daily doses of biscuits supplemented with an olive pomace extract (8.11 µg of HT/g biscuit, provides 729.9 µg of HT), consumed daily for eight weeks, increased homovanillic acid and DOPAC levels, which were suggested by the authors to be involved in the reduction in oxidative LDL cholesterol. Note, however, that sensory changes may occur, as noticed by Navarro and Morales [93] in HT and OLE-fortified biscuits (at different concentrations of HT, 2.55, 5.11 and 10.22 mg of HT/g dough, and OLE, 0.127 and 0.537 mg of OLE/g dough), which became darker than the plain products. Yet, in some conditions, a synergy between components of the extract can contribute to the antiglycative effect; hence, suppressing these changes. A similar pattern of results was obtained in the work of Cedola et al. [94]. In the Italian biscuit “taralli”, the replacement of the ingredient white wine by OLE (17% weight/weight, 24.08 mg gallic acid equivalent/g dry weight) turned the biscuit color darker without changing the overall quality score. Moreover, the addition of OLE improved the nutritional quality of “taralli”, increasing antioxidant activity, total phenols and, especially, flavonoid content from 0.09 mg quercetin/g dry weight in plain samples to 0.39 and 0.36 mg quercetin/g dry weight of uncooked and cooked, respectively [94].

Apart from this, one must also remark the promising application of HT as a functional ingredient in bakery products, aiming to improve their quality. In this regard, the fortification of bread and rusk with olive polyphenols by emulsion was reported to extend their shelf-life from 10 to 15 days. Among the tested concentrations (50–3000 mg olive phenols/kg bread, 100–400 mg olive phenols/kg rusk), that of 200 mg of polyphenols/kg was the most efficient in terms of antimicrobial activity, particularly in rusk [95]. Furthermore, related to this topic, a bread containing Hytolive 2 developed from Genosa (Spain), and the Puratos’ Nostrum brand bread is possible to find on the market [96]. However, in vivo tests are needed to support the potential of these breads as a functional food.

#### 3.3.2. Animal-Based Food Products

Animal-based products such as meat, fish and dairy products [97,98,99,100,101,102,103,104,105,106,107,108,109,110,111,112,113,114,115,116,117,118,119], using HT as an ingredient, are summarized in Table 2.

##### Meat-Based Products 

Meat products with high-fat content, such as lard or sausages, are prone to lipid oxidation either during manufacturing, storage or cooking, and this may lead to a decline in nutritional quality, color changes, texture deterioration, off-odors and off-flavors, and generation of toxic compounds [97]. Concerns about the negative health impacts of the industrial use of nitrites and synthetic antioxidants, such as BHT or BHA [98,99,100], have increased over the last few years and they have clearly prompted the search for natural antioxidant alternatives, such as HT. The fortification of HT in meat-based products has been reported by several authors (Table 2), most of them already considered in the review of Martínez et al. [101].

In this context, Cofrades et al. [102] studied the antioxidant activity of HT-fortified sausage frankfurters with 100 mg of HT/kg meat batter, highlighting that HT effectively inhibited oxidation to the same extent of BHA and BHT. A smaller amount was applied by Nieto et al. [103], 50 mg of HT/kg sausages, which still prevented the lipid oxidation and the loss of thiol groups, in comparison with control samples. The authors suggested that the replacement of animal fat by olive oil and walnuts could be an alternative to produce healthier meat products. Similar antioxidant potential was noticed with the application of 150 mg OMWW extract/kg of lard (HT representing 66.8%) [104], as well through the use of a purified phenolic-rich extract obtained from olive vegetation water in fresh pork sausages [105]. In the same line, in lamb meat patties enriched with omega-3 (*n*-3) fatty acids, the addition of the olive waste extract Hytolive^®^ (containing 10.5% of HT), at concentrations of 100, 200 or 400 mg gallic acid equivalent/kg muscle, was demonstrated to delay meat discoloration, lipid oxidation, and protein carbonylation, and increased the loss of thiol groups relative to controls, during six days of storage [106]. 

Nevertheless, Nieto et al. [103] reported changes in the general acceptability of the sausages supplemented with olive oil and HT-rich extract (50 mg/kg), mostly in flavor and odor, especially when supplemented with olive oil, which lowered the sensorial acceptability score. Yet, the opposite trend was observed by Aquilani et al. [107] and Chaves-López et al. [108] in HT-fortified fermented sausages under different concentrations (higher, around 11.65 mg HT/kg) or even using a distinct supplementation method (submersion in 2.5% of OMWW extract), for which the authors only noticed a color change. Additionally, it was also pointed out that the HT supplementation resulted in a more compact meat emulsion, less porous structure, and more reddish color as a consequence of the possible interaction of HT with fat and protein particles with myoglobin [103]. 

In the context of in vivo experiments supporting the health-promoting effects caused by HT-fortified meat products, Santos-López et al. [109] studied the impact on pork meat supplemented with 3.6 g HT/kg of fresh batter on the lipoprotein profile of aged rats fed high cholesterol/high saturated fat diets. The results show a decrease in adverse effects associated with the diet, mainly by reducing the amount of total cholesterol.

In addition to preventing lipid oxidation, HT-rich extracts have also been claimed to be able to inhibit the growth of major foodborne pathogens, such as *Escherichia coli*, *Listeria monocytogenes*, *Staphylococcus* spp., *Clostridium* spp. and *Salmonella* spp. This effect was comparable to sodium nitrite, i.e., a commercial preservative usually used in meat products [107]. In the study of Chaves-López et al. [108], fermented sausages dipped in 2.5% of OMWW extract (100.23 mg HT/g of extract) resisted the development of fungal species and volatile compound characteristics of fatty acid oxidation. In the work of Rounds and colleagues [110], the authors demonstrated that the addition of an olive oil extract to ground beef (1 and 3%) reduced the *E. coli* population to levels below detectable limits, as well as the amine formation (i.e., carcinogenic compounds that resulted from the heating process) to about half of their initial values. Moreover, the application of olive leaf extracts from *Olea europaea* var. sylvestris (rich in oleuropein and HT) to raw halal minced beef at 1 and 5% was shown to improve the levels of antioxidants in the food products, and simultaneously reduce psychrotrophic counts and pathogens, without any influence on the overall acceptability [111]. 

More recently, the effect of a HT-rich extract from vegetative waters on the shelf-life of lamb meat burger patties was tested [112], allowing the conclusion that the fortification of this product in 200 mg/kg patties prevented lipid oxidation and microbiological growth, although with less efficiency when compared to pure HT.

##### Fishery-Based Products

In addition to meat products, fishery products are a great source of lipids, particularly of long-chain polyunsaturated fatty acids ω-3 (PUFAs), which makes them well recommended by the World Health Organization for preventing cardiovascular diseases. Within this scope, their preservation becomes essential and many antioxidant compounds have been tested to prevent oxidative deterioration of PUFAs during processing and storage. Curiously, the fortification of horse mackerel, bulk cod liver oil and cod liver oil-in-water emulsions with HT (50 mg of HT/kg) showed comparable results with the propyl gallate (a synthetic phenol) (Table 2) regarding the prevention of lipid oxidation, while in cod liver bulk oil and cod liver oil-in-water emulsions, the best oxidative stability was obtained at 100 mg of HT/kg. In horse mackerel muscle, the peroxide values stayed below 20 milliequivalent (meq) oxygen/kg fat even after four weeks of frozen storage (control showed 100 meq oxygen/kg fat). Furthermore, HT allowed good preservation of the original content of docosahexaenoic acid (DHA, 22:6ω-3), as well as of α-tocopherol (decrements are often used as a reflection of oxidative stress of fish) [113]. Moreover, the application of an OLE was effective to hinder the microbial growth of psychrophilic bacteria in anchovy fillets [114], as well to preserve fish patties against *Staphilococcus aureus*, *E. coli,* and *L. monocytogenes* [115], simultaneously contributing favorable sensory and preventive oxidation properties.

##### Dairy Products 

Consumers of a wide range of ages consume dairy products, which make them promising functional foods. A functional milk beverage (yogurt-like) was developed by mixing an OMWW extract in pasteurized cow’s milk concentrated to a final concentration of 100–200 mg/L (expressed in HT equivalent) (Table 2). The added phenols did not interfere either with the fermentation nor with functional lactic acid bacteria, and the HT content increased during the storage. In fact, the authors claimed that, in terms of phenolic content, the consumption of 100 mL of this beverage is almost equivalent to 20 g of VOO (containing 500 mg/kg phenols), and hence extends the health benefits of olive phenols to a milk beverage [116]. Curiously, a HT-rich product obtained from olive fruits (Medoliva©, 0.5 g HT/g powder) added to yogurt was shown to improve the growth of lactic acid bacteria and to contribute to preventing spoilage during fermentation, as well as to promote benefits towards the lipid metabolism of consumers. In non-declared pathology individuals, the daily consumption of two yogurts (200 g each) for two weeks did not change blood redox status but reduced LDL cholesterol [117]. Thus, this yogurt may be integrated into the concept of functional food. In fact, another HT-fortified yogurt (HT concentration ranges of 0.1 and 0.01%) was developed and patented by Villanova and her collaborators [120], which suggests some competition regarding this type of food.

In addition to yogurt, the application of HT in regular milk has also been reported before. In this regard, Fei et al. [118] described that the addition of an olive oil extract (at concentrations of 0.625 mg/mL, containing ≥ 6% HT) exhibited antimicrobial activity, decreasing the vegetative cells to undetectable levels. In milk or egg-based mayonnaise, the addition of VOO also showed bactericidal activity for *Salmonella enteritidis* and *L*. *monocytogenes* [119], which may be relevant considering mayonnaise’s application in raw products, such as salads. 

## 4. Conclusions

The vastness of information regarding bioactivity, metabolism, and absorption of HT makes it an exciting compound to be considered as a potential functional ingredient. The valorization of HT towards diet improvement is an excellent opportunity to use it as a source of natural antioxidants to replace (or reduce) synthetic additives. Among bioactive properties, HT has been claimed to activate endogenous defense systems, such as the antioxidant enzymes that control and regulate the detoxifying mechanism of mitochondrial biogenesis. So far, clinical studies have been focused on HT ingestion together with olive oil, hindering possible conclusions on the real potential of HT. Additionally, information related to the metabolization process and absorption of the compound is still scarce. Although the bioactivity of some HT metabolites is known, further studies are required to investigate their potential applications.

A great diversity of food products supplemented with HT have been reported. However, the impact of the food matrix on the delivery of HT requires further studies. As HT may provide a bitter taste to food, a sensorial analysis should be considered more often to predict the acceptability of consumers to HT supplementation. The costs of the process of HT recovery, pure or in extracts, and the overall impact of HT-fortification on the price of new HT-enriched food products are also important factors to be considered, in order to obtain a fair balance between the valorization of the product and the acquisition price.

## Figures and Tables

**Table 1:** Applicability of free or extract-based hydroxytyrosol (HT) on edible oils, beverages, and vegetable-based and bakery food products.

Formulation	Dosage Applied to Food Products	Main Findings	Ref
***Edible oils***			
HT Rosemary EXT	200 mg/kg HT in *Echium* oil	↑ oil antioxidant status (1.4-fold) HT > rosemary EXT	[69]
Olive cake/dried *Thymus zygis EXT*	VOO supplementation Three oils doses tested (25 mL): 0.01 ^a^, 0.12 ^b^ and 0.21 ^c^ mg of HT	Thirty-three hypercholesterolemic individuals ingested VOO ^a^, olive EXT supplemented VOO ^b^ and olive/thyme EXT supplemented VOO ^c^ (25 mL of olive oil/day, 3 weeks): ↑ HDL antioxidant compounds; ↑ blood plasma antioxidant activity; No consequences on levels of fat intake	[70]
OMWW EXTHT	200 mg/kg of HT and EXT in husk and olive oils (EXT with 1225.6 mg HT/L)	↓ oxidation rate; ↓ peroxide values HT antioxidant effect > BHT	[71]
Olivefen^®^ DHPG, HT, and α-tocopherol mixes	8, 20, 40 and 80 mg Olivefen^®^ to 50 g vegetable oil; Olivefen (37.75% HT) Mixes (0.125–3 mmol/kg vegetable oil)	↑ oxidative stability of oils (DHPG/HT combination > Olivefen^®^); Antioxidant synergic effect in HT/DHPG mixtures	[72]
OMWW EXT	350 to 531 mg HT/kg refined olive oil for French fries	↓ α-tocopherol oxidation; ↓ formation acrolein and hexanal Preservation of French fries sensory and nutritional values; ↑ color, texture score and taste	[73]
Olive cake EXT	Olive oil supplementation Olive cake EXT = 6.64 mg HT/kg olive oil cake	Single-dose of 30 mL enriched olive oil administered to thirteen pre- and stage-1 hypertensive patients: ↓ oxidation of LDL; more benefits on endothelial function than a standard VOO - ↑ ischemic reactive hyperemia	[74]
PO + gallic acid, caffeic acid and HT	PO/EVO: 20:80, 40:60 and 80:20 (100 mg each phenol /kg PO)	No PO oxidation stability by HT (gallic acid > caffeic acid > HT); PO stabilize mixtures < 20% EVO	[75]
HT	Patented application of HT to supplements and edibles oils 30–300 mg HT/kg edible oils 300–30,000 mg HT/kg dietary supplements	Prevention/treatment of cardiovascular diseases, plaque build-up, arterial hypertension, metabolic syndrome	[76]
***Beverages***			
Olive by-products HT EXT	80 mg HT or EXT/L white wine (Sauvignon blanc)	↑ yellowness; ↑ visual appearance score (first six months of bottling); preservation of VC; ↓ Sauvignon blanc wines character (≠ aromas)	[80]
Olive by-products HT EXT	50 mg HT or EXT/L red wine (Syrah)	better red wine chromatic parameters (at bottling); ↓ fruit aroma scents and oxidation odors; ↓ Syrah characteristics after six months	[82]
Olive leaf EXT (OLE)	30 and 60 mg OLE/100 mL fruit smoothies “strawberry-banana”	↑ bitterness perception; recommended < 20 mg OLE/100 g (to prevent bitterness)	[83]
HT	0.02, 0.2 and 1 mg HT/mL tomato juice	0.2 and 1 mg/mL of HT ↑ antioxidant activity (3 and 8-fold, respectively); 1 mg/mL of HT ↓ lipid peroxidation (> BHA); No changes in sensory quality and color. Favorable olive oil taste.	[84]
Olive leaves (dry, infusion, atomized extract)	0.34,1.01, 1.74 and 3 g atomized/L; 0.34, 1.01 and 132 mL infusion/L, 9.9 g leaves/L dry leaves (1.27 mg HT/g), infusion (3.43 mg HT/g), and atomized extract (5.58 mg HT/g)	↑ PC, antioxidant, HT content; Sour/astringent taste, herbal aroma; colloidal instability of beer	[85]
***Vegetable-based products***			
OLE	Table olives fermentation (0.7 kg of olives and 0.7 L of brine solution with 200 mg OLE/L)	↑ antioxidant, anti-inflammatory, and antimicrobial substances; ↓ bitter taste; No adverse effect on their sensorial qualities	[88]
OLE	30 kg table olives for 30 L OLE with 8% NaCl brine solution and starter culture	↑ PC, mostly HT (1700 mg/kg flesh olives vs. 900 mg/kg control olives)	[89]
HT/OLEUR EXT	Table olives submerged in EXT (200 mg HT/kg EXT)	↑ HT content in table olive (↑ 109%); ↑ bitterness; good overall acceptability	[87]
OMWW EXT	0.44, 1.00, 2.25 or 5.06 g EXT/kg vegetable meals: bean purée, potato purée, tomato juice	↑ PC (from 3.7 to 13%); ↑ bitterness, sourness, astringency and pungency	[90]
***Bakery products***			
HT	5.25 mg HT/30 g Biscuit (20% of HT is lost during baking)	↓ oxidized LDL levels; No changes in antioxidant activity of human serum (pharmacokinetic assay)	[91]
Olive pomace EXT*	Biscuit enriched in olive pomace extract (PreBiÒ^®^) (8.11 µg HT/g biscuit),	Human intake study (90 g biscuit/day, 8 weeks): subtle change on bacteria abundance of gut microbiota ↑ homovanillic acid and DOPAC; ↓ oxidative LDL cholesterol	[92]
HT/OLE	2.55, 5.11 and 10.22 mg of HT/g dough; 0.127 and 0.537 mg of OLE/g dough OLE (0.15 mg HT/mL)	↑ slight the darkens the biscuits; ↑ antiglycative effect	[93]
OLE	17% OLE (weight/weight) OLE (24.08 mg gallic acid equivalent/g dry weight)	↑ antioxidant activity, total flavonoids and PC ↑ dark color	[94]
OMWW EXT Ascorbic acid α-Tocopherol (powder and emulsion)	50, 200, 500, 1000, 2000, 3000 mg olive PC/kg bread 100, 200, 400 mg olive PC/kg Rusk	200 mg PC/kg was the most efficient as antimicrobial; ↑ antimicrobial emulsification effect and shelf life of both bread (from 10 to 15 days) and rusk samples (up to 12 weeks)	[95]

*Abbreviations:* BHA—3-*t*-butyl-4-hydroxyanisole; BHT—2,6-di-*t*-butyl-4-methylphenol; DHPG—3,4-dihydroxyphenylglycol; DOPAC—3,4-dihydroxyphenylacetic acid; EVO—extra virgin oil; EXT—extract; HDL—high-density lipoprotein; HT—hydroxytyrosol; LDL—low-density lipoproteins; OLE—olive leaf extract; OMWW—olive mill wastewater; PC—phenolic compounds; PO—palm oil; VC—volatile compounds; VOO—virgin olive oil. (^a^—dose of VOO containing 0.01 mg of HT; ^b^—dose of olive EXT supplemented with VOO with 0.12 mg of HT; ^c^— dose of olive/thyme EXT supplemented with VOO with 0.21 mg of HT; ↑—increase; ↓—decrease).

**Table 2:** Applicability of free or extract-based HT on animal-based products and its effect on product quality.

Formulation	Dosage Applied to Food Products	Main Findings	Ref
***Meat products***			
Olive EXT	100 mg HT/kg low-fat frankfurters sausages	↓ lipid oxidation; HT antioxidant potential > BHA/BHT	[102]
OMWW EXT/OLE/Olive oil, walnut	50 mg of HT/kg chicken sausages	↓ lipid and protein oxidation; ↓ sausage acceptability with olive oil. ↓ stale odor of sausages with OMWW EXT and walnuts (7 and 14 days); ↑ cooking loss, change the color, texture, and taste	[103]
OMWW EXT	150 mg OMWW (66.8% of HT)/kg lard	↓ peroxidation	[104]
Olive vegetative water EXT	0.075 and 0.15 g EXT/100 g pork sausage	↓ pH, diacylglycerols, peroxide value and cholesterol oxidation; no unpleasant sensory properties	[105]
Olive waste EXT (Hytolive^®^)	Hytolive^®^: 10.5% HT in EXT 100, 200 or 400 mg GAE/kg lamb meat patties	↓ lipid oxidation and protein carbonylation; ↑ loss of thiol groups during 6 days of storage; ↓ meat discoloration; change in odor and flavor; texture stable	[106]
Olive pomace HT with grape seed or chestnut EXT	10 g EXT/kg sausages (EXT with11.65 g of HT/L)	↓ lipid oxidation; ↓ microbial growth, comparable to nitrites Changes in profile, color, and texture; good acceptability	[107]
OMWW EXT	100.23 ± 5.25 mg HT/g EXT Fermented sausages dipped 2.5% of OMWW	↓ undesired fungal species growth; ↓ VC (from fatty acids oxidative degradation)	[108]
Olive oil EXT	Olive oil, 1 and 3% to ground beef	↓ E. coli cell count; ↓ amine formation	[110]
HT	3.6 g HT/kg fresh pork	Aged rats fed with high cholesterol/high saturated fat diets: ↓ body weight; ↓ cholesterol content in very-low-density lipoprotein (VLDL) (74% less); ↓ VLDL total mass (63% less)	[109]
OLE	1, and 5% of OLE with 7.32% of HT	↑ antioxidant activity; ↓ microbial growth no impact on overall acceptability	[111]
HT and OMWW extract	200 mg/kg lamb burgers patties HT 99.2% purity; OMWW extract: 7.3% HT	Both induced ↓ lipid oxidation and ↓ the microbiological growth; good color maintenance and general acceptability; ↑ loss of thiol maintenance and general acceptability. HT more efficient	[112]
***Fishery products***			
HT	50 and 100 mg HT/kg fresh horse mackerel (*Trachurus trachurus*), cod (*Gadus morhua*) liver oil	↓ lipid oxidation in bulk oil and minced horse mackerel during frozen storage; ↓ generation of oxidation products; preservation of PUFAs content	[113]
OLE	Anchovy fillets: marinade (1:1) marinade = 10 mg/mL of OLE	in vitro antimicrobial activity, inclusive psychrophilic bacteria ↓ oxidation; preservation of texture, appearance, and organoleptic characteristics	[114]
OLE Fruit EXT	200 mg OLE/kg fish patties	↑ antioxidant activity; ↓ VC (oxidation indicators); ↓ viable count patties, after 11 days (*S. Aureus > E. coli* and *L. monocytogenes*)	[115]
***Dairy products***			
OMWW EXT	100 or 200 mg PC/mL functional milk beverage (yogurt-like)	No interference with the fermentation/lactic acid bacteria; No impact on the VC	[116]
Medoliva© (Polyhealth S.A. Larissa, Greece)	olive PC encapsulated in maltodextrin (0.5 mg HT/g powder) Greek yogurt 2% fat	Human intake of 50 mg olive PC + 400 g yogurt/day, 2 weeks: ↓ body weight, body mass index, hip circumference and systolic blood pressure; ↓ LDL cholesterol levels In yogurt: ↑ lactic acid bacteria during fermentation, ↓ spoilage	[117]
Olive oil extract	0.625 mg EXT/mL milk	↓ vegetative cells to undetectable levels	[118]
VOO EXT Vinegar Wine	VOO (52.8 mg HT/kg); olive oil (14.1 mg HT/kg) VOO EXT (36.9 mg HT/kg); olive oil EXT (13.5 mg HT/kg to milk and egg mayonnaises	↑ bactericidal effect (vinegar > aqueous VOO EXT > wines > olive oil EXT); ↓ *Salmonella Enteritidis and Listeria monocytogenes* (VOO mayonnaise ~ 3 log CFU/g > olive oil mayonnaise)	[119]

*Abbreviations:* BHA—3-*t*-butyl-4-hydroxyanisole; BHT—2,6-di-*t*-butyl-4-methylphenol; CFU—colony-forming unit; EXT—extract; GAE—gallic acids equivalent; HT—hydroxytyrosol; LDL—low-density lipoproteins; OLE—olive leaf extract; OMWW—olive mill wastewater; PC—phenolic compounds; PUFAs—polyunsaturated fatty acids; VC—volatile compounds; VLDL—very-low-density lipoprotein; VOO—virgin olive oil. (↑—increase; ↓—decrease).

## References

[1] Turck D., Bresson J., Burlingame B., Dean T., Fairweather-Tait S., Heinonen M., Hirsch-Ernst K.I., Mangelsdorf I., McArdle H.J., Naska A. (2017). Safety of hydroxytyrosol as a novel food pursuant to Regulation (EC) No 258/97. EFSA J..

[2] (2019). Nova Mentis GRAS Notice (GRN) No. 876. Office of Food Additive Safety. https://www.fda.gov/media/134474/download.

[3] (2017). Europeo COMMISSION IMPLEMENTING DECISION (EU) 2017/2373 of 14 December 2017 authorising the placing on the market of hydroxytyrosol as a novel food ingredient under Regulation (EC) No 258/97 of the European Parliament and of the Council. Off. J. Eur. Union.

[4] Parliament E., States M. (2012). COMMISSION REGULATION (EU) No 432/2012 of 16 May 2012 establishing a list of permitted health claims made on foods, other than those referring to the reduction of disease risk and to children’s development and health. Off. J. Eur. Union.

[5] Boskou D., Boskou D. (2008). Phenolic Compounds in Olives and Olive Oil. Olive oil:Minor Constituents and Health.

[6] García-García M.I., Hernández-García S., Sánchez-Ferrer Á., García-Carmona F. (2013). Kinetic Study of Hydroxytyrosol Oxidation and Its Related Compounds by Red Globe Grape Polyphenol Oxidase. J. Agric. Food Chem..

[7] Fernández-Mar M.I., Mateos R., García-Parrilla M.C., Puertas B., Cantos-Villar E. (2012). Bioactive compounds in wine: Resveratrol, hydroxytyrosol and melatonin: A review. Food Chem..

[8] Cardoso S.M., Guyot S., Marnet N., Lopes-da-Silva J.A., Renard C.M.G.C., Coimbra M.A. (2005). Characterisation of phenolic extracts from olive pulp and olive pomace by electrospray mass spectrometry. J. Sci. Food Agric..

[9] Ramírez E., Brenes M., García P., Medina E., Romero C. (2016). Oleuropein hydrolysis in natural green olives: Importance of the endogenous enzymes. Food Chem..

[10] Santos M.M., Piccirillo C., Castro P.M.L., Kalogerakis N., Pintado M.E. (2012). Bioconversion of oleuropein to hydroxytyrosol by lactic acid bacteria. World J. Microbiol. Biotechnol..

[11] Di Tommaso D., Calabrese R., Rotilio D. (1998). Identification and Quantitation of Hydroxytyrosol in Italian Wines. J. High. Resolut. Chromatogr..

[12] Domínguez-Avila J.A., Wall-Medrano A., Velderrain-Rodríguez G.R., Chen C.-Y.O., Salazar-López N.J., Robles-Sánchez M., González-Aguilar G.A. (2017). Gastrointestinal interactions, absorption, splanchnic metabolism and pharmacokinetics of orally ingested phenolic compounds. Food Funct..

[13] Pereira-Caro G., Sarriá B., Madrona A., Espartero J.L., Escuderos M.E., Bravo L., Mateos R. (2012). Digestive stability of hydroxytyrosol, hydroxytyrosyl acetate and alkyl hydroxytyrosyl ethers. Int. J. Food Sci. Nutr..

[14] Miro-Casas E. (2003). Hydroxytyrosol Disposition in Humans. Clin. Chem..

[15] Robles-Almazan M., Pulido-Moran M., Moreno-Fernandez J., Ramirez-Tortosa C., Rodriguez-Garcia C., Quiles J.L., Ramirez-Tortosa M.C. (2018). Hydroxytyrosol: Bioavailability, toxicity, and clinical applications. Food Res. Int..

[16] Mateos R., Pereira-Caro G., Saha S., Cert R., Redondo-Horcajo M., Bravo L., Kroon P.A. (2011). Acetylation of hydroxytyrosol enhances its transport across differentiated Caco-2 cell monolayers. Food Chem..

[17] Rubió L., Macià A., Valls R.M., Pedret A., Romero M.-P., Solà R., Motilva M.-J. (2012). A new hydroxytyrosol metabolite identified in human plasma: Hydroxytyrosol acetate sulphate. Food Chem..

[18] De La Cruz J.P., Ruiz-Moreno M.I., Guerrero A., López-Villodres J.A., Reyes J.J., Espartero J.L., Labajos M.T., González-Correa J.A. (2015). Role of the catechol group in the antioxidant and neuroprotective effects of virgin olive oil components in rat brain. J. Nutr. Biochem..

[19] Raneva V., Shimasaki H., Ishida Y., Ueta N., Niki E. (2001). Antioxidative activity of 3,4-dihydroxyphenylacetic acid and caffeic acid in rat plasma. Lipids.

[20] Deiana M., Incani A., Rosa A., Corona G., Atzeri A., Loru D., Paola Melis M., Assunta Dessì M. (2008). Protective effect of hydroxytyrosol and its metabolite homovanillic alcohol on H2O2 induced lipid peroxidation in renal tubular epithelial cells. Food Chem. Toxicol..

[21] Gordon M.H., Paiva-Martins F., Almeida M. (2001). Antioxidant Activity of Hydroxytyrosol Acetate Compared with That of Other Olive Oil Polyphenols. J. Agric. Food Chem..

[22] González-Molina E., Moreno D.A., García-Viguera C. (2008). Aronia-Enriched Lemon Juice: A New Highly Antioxidant Beverage. J. Agric. Food Chem..

[23] Burke W.J., Li S.W., Williams E.A., Nonneman R., Zahm D.S. (2003). 3,4-Dihydroxyphenylacetaldehyde is the toxic dopamine metabolite in vivo: Implications for Parkinson’s disease pathogenesis. Brain Res..

[24] López de las Hazas M.C., Piñol C., Macià A., Romero M.P., Pedret A., Solà R., Rubió L., Motilva M.J. (2016). Differential absorption and metabolism of hydroxytyrosol and its precursors oleuropein and secoiridoids. J. Funct. Foods.

[25] Pastor A., Rodríguez-Morató J., Olesti E., Pujadas M., Pérez-Mañá C., Khymenets O., Fitó M., Covas M.-I., Solá R., Motilva M.-J. (2016). Analysis of free hydroxytyrosol in human plasma following the administration of olive oil. J. Chromatogr. A.

[26] González-Santiago M., Fonollá J., Lopez-Huertas E. (2010). Human absorption of a supplement containing purified hydroxytyrosol, a natural antioxidant from olive oil, and evidence for its transient association with low-density lipoproteins. Pharmacol. Res..

[27] Tuck K.L., Freeman M.P., Hayball P.J., Stretch G.L., Stupans I. (2001). The In Vivo Fate of Hydroxytyrosol and Tyrosol, Antioxidant Phenolic Constituents of Olive Oil, after Intravenous and Oral Dosing of Labeled Compounds to Rats. J. Nutr..

[28] Tsimidou M.Z., Nenadis N., Servili M., García-González D.L., Gallina Toschi T. (2018). Why Tyrosol Derivatives Have to Be Quantified in the Calculation of “Olive Oil Polyphenols” Content to Support the Health Claim Provisioned in the EC Reg. 432/2012. Eur. J. Lipid Sci. Technol..

[29] Bulotta S., Celano M., Lepore S.M., Montalcini T., Pujia A., Russo D. (2014). Beneficial effects of the olive oil phenolic components oleuropein and hydroxytyrosol: Focus on protection against cardiovascular and metabolic diseases. J. Transl. Med..

[30] Echeverría F., Ortiz M., Valenzuela R., Videla L. (2017). Hydroxytyrosol and Cytoprotection: A Projection for Clinical Interventions. Int. J. Mol. Sci..

[31] Bertelli M., Kiani A.K., Paolacci S., Manara E., Kurti D., Dhuli K., Bushati V., Miertus J., Pangallo D., Baglivo M. (2020). Hydroxytyrosol: A natural compound with promising pharmacological activities. J. Biotechnol..

[32] Kitsati N., Mantzaris M.D., Galaris D. (2016). Hydroxytyrosol inhibits hydrogen peroxide-induced apoptotic signaling via labile iron chelation. Redox Biol..

[33] Visioli F., Bellomo G., Galli C. (1998). Free Radical-Scavenging Properties of Olive Oil Polyphenols. Biochem. Biophys. Res. Commun..

[34] O′Dowd Y., Driss F., Dang P.M.-C., Elbim C., Gougerot-Pocidalo M.-A., Pasquier C., El-Benna J. (2004). Antioxidant effect of hydroxytyrosol, a polyphenol from olive oil: Scavenging of hydrogen peroxide but not superoxide anion produced by human neutrophils. Biochem. Pharmacol..

[35] Rietjens S.J., Bast A., Haenen G.R.M.M. (2007). New Insights into Controversies on the Antioxidant Potential of the Olive Oil Antioxidant Hydroxytyrosol. J. Agric. Food Chem..

[36] Peng S., Zhang B., Yao J., Duan D., Fang J. (2015). Dual protection of hydroxytyrosol, an olive oil polyphenol, against oxidative damage in PC12 cells. Food Funct..

[37] Crupi R., Palma E., Siracusa R., Fusco R., Gugliandolo E., Cordaro M., Impellizzeri D., De Caro C., Calzetta L., Cuzzocrea S. (2020). Protective Effect of Hydroxytyrosol Against Oxidative Stress Induced by the Ochratoxin in Kidney Cells: In vitro and in vivo Study. Front. Vet. Sci..

[38] Goya L., Mateos R., Bravo L. (2007). Effect of the olive oil phenol hydroxytyrosol on human hepatoma HepG2 cells. Eur. J. Nutr..

[39] Burattini S., Salucci S., Baldassarri V., Accorsi A., Piatti E., Madrona A., Espartero J.L., Candiracci M., Zappia G., Falcieri E. (2013). Anti-apoptotic activity of hydroxytyrosol and hydroxytyrosyl laurate. Food Chem. Toxicol..

[40] Aldini G., Piccoli A., Beretta G., Morazzoni P., Riva A., Marinello C., Maffei Facino R. (2006). Antioxidant activity of polyphenols from solid olive residues of c.v. Coratina. Fitoterapia.

[41] Vlavcheski F., Young M., Tsiani E. (2019). Antidiabetic Effects of Hydroxytyrosol: In Vitro and In Vivo Evidence. Antioxidants.

[42] Zhu L., Liu Z., Feng Z., Hao J., Shen W., Li X., Sun L., Sharman E., Wang Y., Wertz K. (2010). Hydroxytyrosol protects against oxidative damage by simultaneous activation of mitochondrial biogenesis and phase II detoxifying enzyme systems in retinal pigment epithelial cells. J. Nutr. Biochem..

[43] Giordano E., Dávalos A., Visioli F. (2014). Chronic hydroxytyrosol feeding modulates glutathione-mediated oxido-reduction pathways in adipose tissue: A nutrigenomic study. Nutr. Metab. Cardiovasc. Dis..

[44] Granados-Principal S., El-azem N., Pamplona R., Ramirez-Tortosa C., Pulido-Moran M., Vera-Ramirez L., Quiles J.L., Sanchez-Rovira P., Naudí A., Portero-Otin M. (2014). Hydroxytyrosol ameliorates oxidative stress and mitochondrial dysfunction in doxorubicin-induced cardiotoxicity in rats with breast cancer. Biochem. Pharmacol..

[45] González-Santiago M., Martín-Bautista E., Carrero J.J., Fonollá J., Baró L., Bartolomé M.V., Gil-Loyzaga P., López-Huertas E. (2006). One-month administration of hydroxytyrosol, a phenolic antioxidant present in olive oil, to hyperlipemic rabbits improves blood lipid profile, antioxidant status and reduces atherosclerosis development. Atherosclerosis.

[46] Fuccelli R., Fabiani R., Rosignoli P. (2018). Hydroxytyrosol Exerts Anti-Inflammatory and Anti-Oxidant Activities in a Mouse Model of Systemic Inflammation. Molecules.

[47] Pan S., Liu L., Pan H., Ma Y., Wang D., Kang K., Wang J., Sun B., Sun X., Jiang H. (2013). Protective effects of hydroxytyrosol on liver ischemia/reperfusion injury in mice. Mol. Nutr. Food Res..

[48] Feng Z., Bai L., Yan J., Li Y., Shen W., Wang Y., Wertz K., Weber P., Zhang Y., Chen Y. (2011). Mitochondrial dynamic remodeling in strenuous exercise-induced muscle and mitochondrial dysfunction: Regulatory effects of hydroxytyrosol. Free Radic. Biol. Med..

[49] Jemai H., Bouaziz M., Fki I., El Feki A., Sayadi S. (2008). Hypolipidimic and antioxidant activities of oleuropein and its hydrolysis derivative-rich extracts from Chemlali olive leaves. Chem. Biol. Interact..

[50] Hamden K., Allouche N., Damak M., Elfeki A. (2009). Hypoglycemic and antioxidant effects of phenolic extracts and purified hydroxytyrosol from olive mill waste in vitro and in rats. Chem. Biol. Interact..

[51] Covas M.-I., Nyyssönen K., Poulsen H.E., Kaikkonen J., Zunft H.-J.F., Kiesewetter H., Gaddi A., de la Torre R., Mursu J., Bäumler H. (2006). The Effect of Polyphenols in Olive Oil on Heart Disease Risk Factors. Ann. Intern. Med..

[52] D’Angelo S., Manna C., Migliardi V., Mazzoni O., Morrica P., Capasso G., Pontoni G., Galletti P., Zappia V. (2001). Pharmacokinetics and metabolism of hydroxytyrosol, a natural antioxidant from olive oil. Drug Metab. Dispos..

[53] Christian M.S., Sharper V.A., Hoberman A.M., Seng J.E., Fu L., Covell D., Diener R.M., Bitler C.M., Crea R. (2004). The Toxicity Profile of Hydrolyzed Aqueous Olive Pulp Extract. Drug Chem. Toxicol..

[54] Auñon-Calles D., Canut L., Visioli F. (2013). Toxicological evaluation of pure hydroxytyrosol. Food Chem. Toxicol..

[55] Achmon Y., Fishman A. (2015). The antioxidant hydroxytyrosol: Biotechnological production challenges and opportunities. Appl. Microbiol. Biotechnol..

[56] Veneziani G., Novelli E., Esposto S., Taticchi A., Servili M. (2017). Applications of recovered bioactive compounds in food products. Olive Mill Waste.

[57] Changping L., Zhuo L. (2017). A Kind of Extracting Process of Hydroxytyrosol. Patent.

[58] Más J.A.L., Mellado M.P., Ortiz P.M., Streitenberger S.A. (2010). Process and Apparatus for the Production of Hydroxytyrosol Containing Extract from Olives and Solids Containing Residues of Olive Oil Extraction. Patent.

[59] Weiping H. (2017). The Method that Hydroxytyrosol Is Extracted from Processing Olive Oil Waste Water. Patent.

[60] Lingxiao, Shende J., Xinyan G., Chao X. (2013). Method for Preparing High-Purity Hydroxytyrosol. Patent.

[61] Milczarek R., Larson D., Li Y.O., Sedej I., Wang S. (2019). Olive. Integrated Processing Technologies for Food and Agricultural By-Products.

[62] Ulm J., Leuenberger B.H. (2009). Novel Powders Based on Vegetation Water from Olive Oil Production. Patent.

[63] Raederstorff D., Richard N., Schwager J., Wertz K. (2010). Compositions. U.S. Patent.

[64] Martínez N., Herrera M., Frías L., Provencio M., Pérez-Carrión R., Díaz V., Morse M., Crespo M.C. (2019). A combination of hydroxytyrosol, omega-3 fatty acids and curcumin improves pain and inflammation among early stage breast cancer patients receiving adjuvant hormonal therapy: Results of a pilot study. Clin. Transl. Oncol..

[65] Carito V., Ciafrè S., Tarani L., Ceccanti M., Natella F., Iannitelli A., Tirassa P., Chaldakov N.G., Ceccanti M., Boccardo C. (2015). TNF-α and IL-10 modulation induced by polyphenols extracted by olive pomace in a mouse model of paw inflammation. Ann. Ist Super Sanità.

[66] Aponte M., Ungaro F., D’Angelo I., De Caro C., Russo R., Blaiotta G., Dal Piaz F., Calignano A., Miro A. (2018). Improving in vivo conversion of oleuropein into hydroxytyrosol by oral granules containing probiotic Lactobacillus plantarum 299v and an *Olea europaea* standardized extract. Int. J. Pharm..

[67] Quirós-Fernández R., López-Plaza B., Bermejo L., Palma-Milla S., Gómez-Candela C. (2019). Supplementation with Hydroxytyrosol and Punicalagin Improves Early Atherosclerosis Markers Involved in the Asymptomatic Phase of Atherosclerosis in the Adult Population: A Randomized, Placebo-Controlled, Crossover Trial. Nutrients.

[68] Kehili M., Choura S., Zammel A., Allouche N., Sayadi S. (2018). Oxidative stability of refined olive and sunflower oils supplemented with lycopene-rich oleoresin from tomato peels industrial by-product, during accelerated shelf-life storage. Food Chem..

[69] Bañares C., Martin D., Reglero G., Torres C.F. (2019). Protective effect of hydroxytyrosol and rosemary extract in a comparative study of the oxidative stability of Echium oil. Food Chem..

[70] Farràs M., Fernández-Castillejo S., Rubió L., Arranz S., Catalán Ú., Subirana I., Romero M.-P., Castañer O., Pedret A., Blanchart G. (2018). Phenol-enriched olive oils improve HDL antioxidant content in hypercholesterolemic subjects. A randomized, double-blind, cross-over, controlled trial. J. Nutr. Biochem..

[71] Fki I., Allouche N., Sayadi S. (2005). The use of polyphenolic extract, purified hydroxytyrosol and 3,4-dihydroxyphenyl acetic acid from olive mill wastewater for the stabilization of refined oils: A potential alternative to synthetic antioxidants. Food Chem..

[72] Lama-Muñoz A., Rubio-Senent F., Bermúdez-Oria A., Fernández-Prior Á., Fernández-Bolaños J., Rodríguez-Gutiérrez G. (2019). Synergistic effect of 3,4-dihydroxyphenylglycol with hydroxytyrosol and α-tocopherol on the Rancimat oxidative stability of vegetable oils. Innov. Food Sci. Emerg. Technol..

[73] Sordini B., Veneziani G., Servili M., Esposto S., Selvaggini R., Lorefice A., Taticchi A. (2019). A quanti-qualitative study of a phenolic extract as a natural antioxidant in the frying processes. Food Chem..

[74] Valls R.-M., Farràs M., Suárez M., Fernández-Castillejo S., Fitó M., Konstantinidou V., Fuentes F., López-Miranda J., Giralt M., Covas M.-I. (2015). Effects of functional olive oil enriched with its own phenolic compounds on endothelial function in hypertensive patients. A randomised controlled trial. Food Chem..

[75] De Leonardis A., Macciola V. (2012). Heat-oxidation stability of palm oil blended with extra virgin olive oil. Food Chem..

[76] Más J.A.L., Streitenberger S.A., Mellado M.P., Ortiz P.M. (2009). Fortification of Nutritional Products with Olive Extracts Containing Hydroxytyrosol and Hydroxytyrosol Fortified Nutritional Products. Patent.

[77] Bulbarello A., Leuthardt B. (2016). Fortification of Edible Oils with Hyrdoxytyrosol. Patent.

[78] De Hierro A.G.-G.L., Sánchez A.P. (2008). Olive Oil Composition and Use Thereof as Functional Food. Patent.

[79] Vally H., Misso N.L.A., Madan V. (2009). Clinical effects of sulphite additives. Clin. Exp. Allergy.

[80] Raposo R., Ruiz-Moreno M.J., Garde-Cerdán T., Puertas B., Moreno-Rojas J.M., Zafrilla P., Gonzalo-Diago A., Guerrero R.F., Cantos-Villar E. (2016). Replacement of sulfur dioxide by hydroxytyrosol in white wine: Influence on both quality parameters and sensory. LWT Food Sci. Technol..

[81] Ruiz-Moreno M.J., Raposo R., Moreno-Rojas J.M., Zafrilla P., Cayuela J.M., Mulero J., Puertas B., Guerrero R.F., Piñeiro Z., Giron F. (2015). Efficacy of olive oil mill extract in replacing sulfur dioxide in wine model. LWT Food Sci. Technol..

[82] Raposo R., Ruiz-Moreno M.J., Garde-Cerdán T., Puertas B., Moreno-Rojas J.M., Gonzalo-Diago A., Guerrero R.F., Ortiz V., Cantos-Villar E. (2016). Effect of hydroxytyrosol on quality of sulfur dioxide-free red wine. Food Chem..

[83] Kranz P., Braun N., Schulze N., Kunz B. (2010). Sensory quality of functional beverages: Bitterness perception and bitter masking of olive leaf extract fortified fruit smoothies. J. Food Sci..

[84] Larrosa M., Espín J.C., Tomás-Barberán F.A. (2003). Antioxidant capacity of tomato juice functionalised with enzymatically synthesised hydroxytyrosol. J. Sci. Food Agric..

[85] Guglielmotti M., Passaghe P., Buiatti S. (2020). Use of olive (*Olea europaea* L.) leaves as beer ingredient, and their influence on beer chemical composition and antioxidant activity. J. Food Sci..

[86] Takashi F., Yuki N., Hideki M. (2017). Beverage Containing Hydroxytyrosol. Patent.

[87] Lalas S., Athanasiadis V., Gortzi O., Bounitsi M., Giovanoudis I., Tsaknis J., Bogiatzis F. (2011). Enrichment of table olives with polyphenols extracted from olive leaves. Food Chem..

[88] Caponio F., Difonzo G., Calasso M., Cosmai L., De Angelis M. (2019). Effects of olive leaf extract addition on fermentative and oxidative processes of table olives and their nutritional properties. Food Res. Int..

[89] Schaide T., Cabrera-Bañegil M., Pérez-Nevado F., Esperilla A., Martín-Vertedor D. (2019). Effect of olive leaf extract combined with Saccharomyces cerevisiae in the fermentation process of table olives. J. Food Sci. Technol..

[90] De Toffoli A., Monteleone E., Bucalossi G., Veneziani G., Fia G., Servili M., Zanoni B., Pagliarini E., Gallina Toschi T., Dinnella C. (2019). Sensory and chemical profile of a phenolic extract from olive mill waste waters in plant-base food with varied macro-composition. Food Res. Int..

[91] Mateos R., Martínez-López S., Baeza Arévalo G., Amigo-Benavent M., Sarriá B., Bravo-Clemente L. (2016). Hydroxytyrosol in functional hydroxytyrosol-enriched biscuits is highly bioavailable and decreases oxidised low density lipoprotein levels in humans. Food Chem..

[92] Conterno L., Martinelli F., Tamburini M., Fava F., Mancini A., Sordo M., Pindo M., Martens S., Masuero D., Vrhovsek U. (2019). Measuring the impact of olive pomace enriched biscuits on the gut microbiota and its metabolic activity in mildly hypercholesterolaemic subjects. Eur. J. Nutr..

[93] Navarro M., Morales F.J. (2017). Effect of hydroxytyrosol and olive leaf extract on 1,2-dicarbonyl compounds, hydroxymethylfurfural and advanced glycation endproducts in a biscuit model. Food Chem..

[94] Cedola A., Palermo C., Centonze D., Del Nobile M.A., Conte A. (2020). Characterization and Bio-Accessibility Evaluation of Olive Leaf Extract-Enriched “Taralli”. Foods.

[95] Galanakis C.M., Tsatalas P., Charalambous Z., Galanakis I.M. (2018). Control of microbial growth in bakery products fortified with polyphenols recovered from olive mill wastewater. Environ. Technol. Innov..

[96] Hayata M., özuğur G. (2011). Phytochemical fortification of flour and bread. Flour and Breads and Their Fortification in Health and Disease Prevention.

[97] Pateiro M., Barba F.J., Domínguez R., Sant’Ana A.S., Mousavi Khaneghah A., Gavahian M., Gómez B., Lorenzo J.M. (2018). Essential oils as natural additives to prevent oxidation reactions in meat and meat products: A review. Food Res. Int..

[98] De Mey E., De Maere H., Paelinck H., Fraeye I. (2017). Volatile N -nitrosamines in meat products: Potential precursors, influence of processing, and mitigation strategies. Crit. Rev. Food Sci. Nutr..

[99] Kawano S., Nakao T., Hiraga K. (1980). Species and Strain Differences in the Butylated Hydroxytoluene (BHT)-Producing Induction of Hepatic Drug Oxidation Enzymes. Jpn. J. Pharmacol..

[100] Park S., Lee J.-Y., Lim W., You S., Song G. (2019). Butylated Hydroxyanisole Exerts Neurotoxic Effects by Promoting Cytosolic Calcium Accumulation and Endoplasmic Reticulum Stress in Astrocytes. J. Agric. Food Chem..

[101] Martínez L., Ros G., Nieto G. (2018). Hydroxytyrosol: Health Benefits and Use as Functional Ingredient in Meat. Medicines.

[102] Cofrades S., Salcedo Sandoval L., Delgado-Pando G., López-López I., Ruiz-Capillas C., Jiménez-Colmenero F. (2011). Antioxidant activity of hydroxytyrosol in frankfurters enriched with n-3 polyunsaturated fatty acids. Food Chem..

[103] Nieto G., Martínez L., Castillo J., Ros G. (2017). Hydroxytyrosol extracts, olive oil and walnuts as functional components in chicken sausages. J. Sci. Food Agric..

[104] De Leonardis A., Macciola V., Lembo G., Aretini A., Nag A. (2007). Studies on oxidative stabilisation of lard by natural antioxidants recovered from olive-oil mill wastewater. Food Chem..

[105] Balzan S., Taticchi A., Cardazzo B., Urbani S., Servili M., Di Lecce G., Zabalza I.B., Rodriguez-Estrada M.T., Novelli E., Fasolato L. (2017). Effect of phenols extracted from a by-product of the oil mill on the shelf-life of raw and cooked fresh pork sausages in the absence of chemical additives. LWT Food Sci. Technol..

[106] Muíño I., Díaz M.T., Apeleo E., Pérez-Santaescolástica C., Rivas-Cañedo A., Pérez C., Cañeque V., Lauzurica S., Fuente J. (2017). de la Valorisation of an extract from olive oil waste as a natural antioxidant for reducing meat waste resulting from oxidative processes. J. Clean. Prod..

[107] Aquilani C., Sirtori F., Flores M., Bozzi R., Lebret B., Pugliese C. (2018). Effect of natural antioxidants from grape seed and chestnut in combination with hydroxytyrosol, as sodium nitrite substitutes in Cinta Senese dry-fermented sausages. Meat Sci..

[108] Chaves-López C., Serio A., Mazzarrino G., Martuscelli M., Scarpone E., Paparella A. (2015). Control of household mycoflora in fermented sausages using phenolic fractions from olive mill wastewaters. Int. J. Food Microbiol..

[109] Santos-López J.A., Garcimartín A., Bastida S., Bautista-Ávila M., González-Muñoz M.J., Benedí J., Sánchez-Muniz F.J. (2018). Lipoprotein Profile in Aged Rats Fed Chia Oil- or Hydroxytyrosol-Enriched Pork in High Cholesterol/High Saturated Fat Diets. Nutrients.

[110] Rounds L., Havens C.M., Feinstein Y., Friedman M., Ravishankar S. (2013). Concentration-dependent inhibition of Escherichia coli O157:H7 and heterocyclic amines in heated ground beef patties by apple and olive extracts, onion powder and clove bud oil. Meat Sci..

[111] Djenane D., Gómez D., Yangüela J., Roncalés P., Ariño A. (2018). Olive Leaves Extract from Algerian Oleaster (*Olea europaea* var. sylvestris) on Microbiological Safety and Shelf-life Stability of Raw Halal Minced Beef during Display. Foods.

[112] Martínez-Zamora L., Ros G., Nieto G. (2020). Synthetic vs. Natural Hydroxytyrosol for Clean Label Lamb Burgers. Antioxidants.

[113] Pazos M., Alonso A., Sánchez I., Medina I. (2008). Hydroxytyrosol Prevents Oxidative Deterioration in Foodstuffs Rich in Fish Lipids. J. Agric. Food Chem..

[114] Testa B., Lombardi S.J., Macciola E., Succi M., Tremonte P., Iorizzo M. (2019). Efficacy of olive leaf extract (*Olea europaea* L. cv Gentile di Larino) in marinated anchovies (*Engraulis encrasicolus*, L.) process. Heliyon.

[115] Martínez L., Castillo J., Ros G., Nieto G. (2019). Antioxidant and Antimicrobial Activity of Rosemary, Pomegranate and Olive Extracts in Fish Patties. Antioxidants.

[116] Servili M., Rizzello C.G., Taticchi A., Esposto S., Urbani S., Mazzacane F., Di Maio I., Selvaggini R., Gobbetti M., Di Cagno R. (2011). Functional milk beverage fortified with phenolic compounds extracted from olive vegetation water, and fermented with functional lactic acid bacteria. Int. J. Food Microbiol..

[117] Georgakouli K., Mpesios A., Kouretas D., Petrotos K., Mitsagga C., Giavasis I., Jamurtas A. (2016). The Effects of an Olive Fruit Polyphenol-Enriched Yogurt on Body Composition, Blood Redox Status, Physiological and Metabolic Parameters and Yogurt Microflora. Nutrients.

[118] Fei P., Xu Y., Zhao S., Gong S., Guo L. (2019). Olive oil polyphenol extract inhibits vegetative cells of Bacillus cereus isolated from raw milk. J. Dairy Sci..

[119] Medina E., Romero C., Brenes M., de Castro A. (2007). Antimicrobial Activity of Olive Oil, Vinegar, and Various Beverages against Foodborne Pathogens. J. Food Prot..

[120] Villanova A., Villanova L., Merendino A., Fasiello G. (2010). Yoghurt Containing Hydroxytyrosol and Other Biophenols with a Preventive Nutritional Activity Beneficial to Human Beings. Patent.

